# An Approach to Steady-State Power Transformer Modeling Considering Direct Current Resistance Test Measurements

**DOI:** 10.3390/s21186284

**Published:** 2021-09-19

**Authors:** Henrique Pires Corrêa, Flávio Henrique Teles Vieira

**Affiliations:** Information and Communication Engineering Group (INCOMM), School of Electrical, Mechanical and Computer Engineering (EMC), Federal University of Goiás (UFG), Goiania 74605-010, Brazil; flavio_vieira@ufg.br

**Keywords:** steady-state model, power transformer, connection resistance

## Abstract

Measurements obtained in transformer tests are routinely used for computing associated steady-state model parameters, which can then be used for load flow simulation and other modeling applications. The short circuit and open circuit tests are most commonly performed with this purpose, allowing estimation of series and parallel branch transformer parameters. In this study, an extended model is proposed which does not employ the usually assumed cantilever circuit approximation and explicitly accounts for transformer connection resistance. An estimation of the proposed model parameters is enabled by usage of additional measurements yielded by the direct current (DC) resistance test. The proposed approach is validated by means of an experiment carried out on a real distribution power transformer, whose results demonstrate that the proposed model and parameter computation approach effectively decompose total transformer resistance into winding and contact components. Furthermore, the numerical results show that contact resistance is not negligible especially for low voltage windings, which reinforces the usefulness of the proposed model in providing detailed modeling of transformer resistances.

## 1. Introduction

Steady-state transformer models are frequently employed in power system load flow simulations and other applications in which component modeling can be carried out in *quasi*-stationary regime [1,2,3,4,5,6,7,8,9]. Different transformer models can be selected according to frequency range [10,11,12] and the required trade-off between precision and complexity. The focus of this work is that of power system load flow modeling, for which reason only low-frequency models are henceforth considered. In this framework, most transformer models are approximate simplifications of the exact one [13], which aim at reducing equivalent circuit complexity and facilitating parameter computation via transformer test measurements. In what follows, the most frequently employed approximate steady-state transformer model, henceforth denoted as the *standard* model, is briefly discussed.

**Figure 1:**
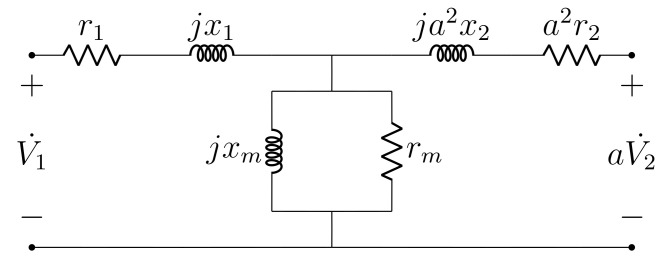
Exact transformer steady-state model.

**Figure 2:**
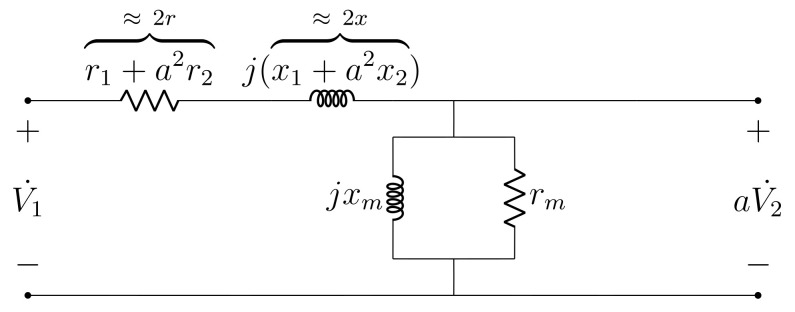
Standard transformer steady-state model.

The exact and standard steady-state alternating current (AC) transformer models are depicted, respectively, in Figure 1 and Figure 2. Parameters $r_{1}$, $x_{1}$, $r_{2}$, $x_{2}$, $g_{m}$, $y_{m}$ and *a* denote: primary resistance, primary reactance, secondary resistance, secondary reactance, core conductance, core susceptance and transformation ratio (assumed as known), respectively. The parameter values are assumed to be given in SI units and referred to the transformer primary, which incorporates the transformation ratio into the model. As seen from Figure 2, the standard model employs the cantilever circuit approximation, which transposes the secondary series impedance to the primary. Furthermore, the assumptions $r=\;r_{1}\approx a^{2}r_{2}$ and $x=\;x_{1}\approx a^{2}x_{2}$ are used to enable parameter computation solely with open circuit and short circuit test measurements [13]. Please note that the exact and standard models are often referred to as, respectively, the T and L models [14].

In this sense, the determination of standard model parameters does not require measurements obtained in the DC resistance test. Nevertheless, such test is also carried out in practice to assess winding integrity [15] and enable computations with respect to the heating of transformer windings at full-load operation [16]. This consideration and transformer testing practice lead to three observations: (a) the DC resistance test is frequently performed in conjunction with open circuit and short circuit tests; (b) it provides additional information regarding $r_{1}$ and $r_{2}$ when compared to the latter tests; and (c) such measurement data can be used for adding detail to the standard model.

Taking such points as motivation, this study proposes a transformer steady-state model which: (a) does not use the cantilever and $r_{1}\approx a^{2}r_{2}$ approximations; (b) includes a parameter that corresponds to connection resistance, which thus becomes separated from the winding resistance; and (c) can be fully determined via measurement results of the DC resistance, short circuit and open circuit tests.

An especially detailed analysis is carried out for the proposed Δ winding model, among which it is shown that an exact solution is attainable if such windings can be temporarily changed to open-Δ connection during the DC resistance test. Since this is usually not feasible in practice (i.e., each winding only has one accessible terminal), an alternative approach is proposed, namely assuming the Δ connection resistance as being equal to a fraction of the winding resistance and, subsequently, optimizing such a fraction.

## 2. Proposed Steady-State Transformer Model

In this section, the proposed model is presented and computation of its parameters by means of transformer test measurements is carried out. At first, DC models for Y and Δ windings are considered. Subsequently, a per phase AC model is given.

### 2.1. Equivalent DC Circuit of Y Winding

The proposed model of a transformer Y winding submitted to the DC resistance measurement test is now described. Please note that this test does not impose balanced conditions to transformer phases, for which reason a per phase approach may not be used. Furthermore, zero frequency implies no induction on the remaining set of windings, which may thus be ignored. For analogous reasons, core parameters and dispersion reactance of the tested windings have no effect. It is assumed that no saturation problems due to residual DC flux affect the transformer [17]. Two measurements are usually made in this test, namely phase-neutral and phase-phase resistances.

**Figure 3:**
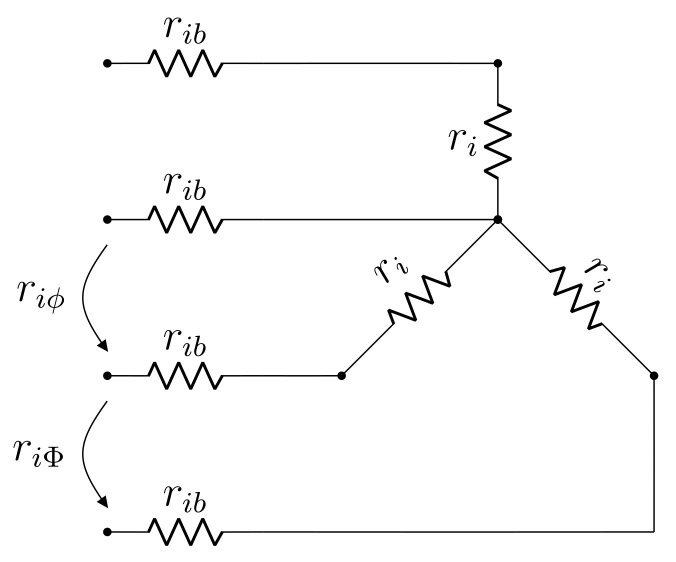
Proposed DC model for Y winding.

Considering the above discussion, the proposed model is depicted in Figure 3, where $r_{i},r_{ib},r_{i\phi }$ and $r_{i\Phi }$ denote, respectively: winding resistance, connection resistance, phase-neutral resistance measurement and phase-phase resistance measurement. Index i∈{1,2} denotes if the winding under consideration is either primary or secondary, respectively. The main innovation of this model is an explicit parameter to account for connection resistance, which may thus be estimated separately from winding resistance. Please note that a fourth $r_{ib}$ element accounts for neutral connection resistance.

Equating input resistances in the model to measured phase-neutral and phase-phase resistance values, a set of two equations is obtained:

(1) $$r_{i}+2r_{ib}=r_{i\phi }$$

(2) $$2r_{i}+2r_{ib}=r_{i\Phi }$$

which may be readily solved for $r_{i}$ and $r_{ib}$. It will be seen that this is not the case for a Δ winding, for which a single equation is obtained with standard measurements. Solving the linear system comprised of Equations (1) and (2), the following is obtained:

(3) $$r_{i}=r_{i\Phi }-r_{i\phi }$$

(4) $$r_{ib}=r_{i\phi }-\frac{1}{2}r_{i\Phi }$$

from which it is clear that all parameters of the proposed Y winding DC model can be determined solely from the DC resistance test measurements.

### 2.2. Equivalent DC Circuit of Δ Winding

**Figure 4:**
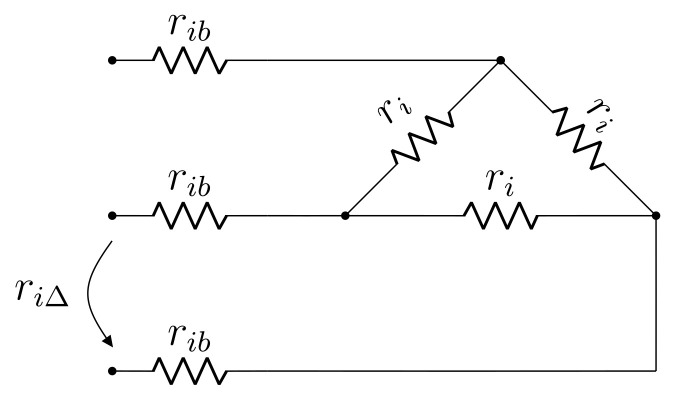
Proposed DC model for Δ winding.

Considerations identical to those made with respect to zero frequency are applicable to the DC resistance test model of a Δ winding. The main difference is the fact that a single measurement is made, namely that of phase-phase resistance. Analogously to the Y winding case, the model in Figure 4 is proposed, in which $r_{i}$, $r_{ib}$ and $r_{i\Delta }$ are, respectively: winding resistance, connection resistance and phase-phase resistance measurement.

**Figure 5:**
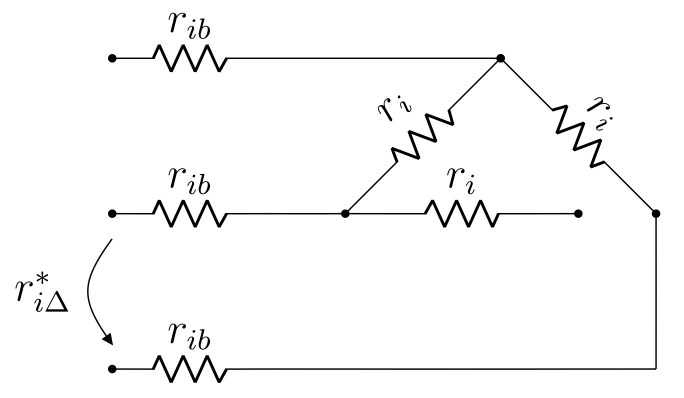
Additional DC measurement for Δ winding.

Equating phase-phase measurement and model input resistance yields:

(5) $$2r_{i}+6r_{ib}=3r_{i\Delta }$$

which is not solvable for both $r_{i}$ and $r_{ib}$. To achieve a solution, an assumption with respect to the Δ side terminals is made, namely that the connection can be temporarily changed to open-Δ [18]. Provided this assumption is true, an additional DC measurement can be made according to Figure 5, from which the following is obtained:

(6) $$2r_{i}+2r_{ib}=r_{i\Delta }^{*}$$

Equations (5) and (6) can be combined to yield the solution:

(7) $$r_{i}=\frac{3}{4}\left(r_{i\Delta }^{*}-r_{i\Delta }\right)$$

(8) $$r_{ib}=\frac{1}{4}\left(3r_{i\Delta }-r_{i\Delta }^{*}\right)$$

In this sense, given the open-Δ assumption, parameters of the proposed Δ winding DC model are readily computed from the DC resistance measurement results.

However, the above-described assumption is usually infeasible due to none of the Δ windings having both of their terminals externally accessible. For this reason, an alternative assumption must be adopted so that a solution may be obtained. It is proposed that $r_{ib}\approx \delta \cdot r_{i}$ be assumed, where 0<δ≪1 is, in principle, a known real number. This approach is motivated by the fact that, provided no contact malfunction exists, connection resistance is expected to be smaller than that of the transformer windings. Proceeding as such and manipulating Equation (5), the following is obtained:

(9) $$r_{i}=\delta^{-1}\cdot r_{ib}=\frac{3}{2+6\delta }r_{i\Delta }$$

If greater accuracy is required, it may be deemed undesirable to assume arbitrary δ. In Section 2.3, a procedure is proposed for optimizing the selection of this parameter.

### 2.3. Equivalent Per-Phase AC Circuit

In what follows, it is assumed that DC resistance measurements have been carried out according to Section 2.1 and Section 2.2. It now remains to incorporate such measurements into an equivalent per phase AC model. To achieve this, it is first noted that resistance values measured in the DC resistance test must be adjusted for full-load operation temperature. Let γ be the resistance temperature coefficient; it is reasonable to assume it as known since it is fixed for the metal of which the conductors are made. The factor by which a DC resistance must be multiplied is:

(10) $$\alpha =1+\gamma (T-T_{DC})$$

where *T* and $T_{DC}$ are conductor temperatures during full-load transformer operation and DC resistance measurement, respectively.

Since connection conductors may be subjected to a different temperature than the actual winding, a multiplicative factor must be computed for each. Keeping in accordance with previous notation, such factors are denoted as α and $\alpha_{b}$ for windings and connections, respectively. If no data are available for computing $\alpha_{b}$, it is reasonable to assume equal winding and connection conductor temperatures by setting $\alpha =\alpha_{b}$.

Please note that additional computations for transformer temperature estimation could be used for greater precision. An interesting alternative consists of modeling the heat transfer process over the transformer structure via electrical circuit analogy [14]. Coupled with data on transformer dimensions, this approach could be used for estimating the temperature distribution over its structure. However, detailed computations are not considered since transformer nameplates provide a rough estimate of the oil temperature increase at full load, which may be deemed sufficient for practical purposes.

**Figure 6:**
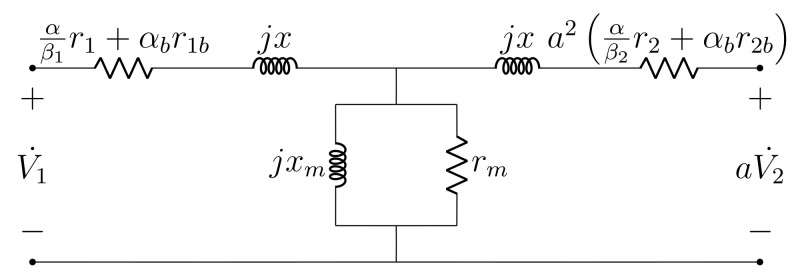
Proposed per phase AC model.

The DC test offers no additional information with respect to dispersion inductances, for which reason the usual assumption $x=x_{1}\approx a^{2}x_{2}$ is kept. The proposed AC model is given in Figure 6, where $\beta_{i}\in \left\{1,3\right\}$, with i∈{1,2}, is used to account for Y and Δ connections, respectively. This is done because impedance values are considered *per winding*, which requires $\beta_{i}=3$ for obtaining the per phase value in Δ connection.

In the open circuit test, behavior of the proposed model is identical to that of the standard model. This is seen by noting that one of the series impedance branches is open-circuited, whereas the remaining one has negligible magnitude with respect to the parallel branch. Hence, $r_{m}$ and $x_{m}$ are determined as usual via this test [13].

Now, consider the short circuit test and let $V_{sc},I_{sc}$ and $P_{sc}$ be, respectively, the measured voltage, current and active power. Recall that the parallel branch may be disregarded in this case due to its large impedance with respect to the short-circuited series branch. At first, *x* may be determined as usual by computing reactive power:

$$2x\cdot I_{sc}^{2}=P_{sc}\cdot \tan\cos^{-1}\left(\frac{P_{sc}}{V_{sc}I_{sc}}\right)$$

(11) $$x=\frac{P_{sc}}{2I_{sc}^{2}}\cdot \tan\cos^{-1}\left(\frac{P_{sc}}{V_{sc}I_{sc}}\right)$$

whereas, given either the open-Δ assumption or the absence of Δ windings, no further computations are required for obtaining $r_{i}$ and $r_{ib}$, i∈{1,2}, which can be determined solely via DC resistance measurements and Equations (1)–(8).

If a Δ winding exists and the alternative assumption $r_{ib}\approx \delta \cdot r_{i}$ is used, a procedure is now proposed for using $P_{sc}$ in order to optimize δ and, as a consequence, the values of $r_{i}$ and $r_{ib}$. Referring to Figure 6 and once more neglecting the parallel branch due to its high impedance, active power can be computed as follows for a Δ-Y connection:

(12) $$\left[\left(\frac{\alpha }{3}+\alpha_{b}\delta \right)r_{1}\left(\delta \right)+a^{2}\left(\alpha r_{2}+\alpha_{b}r_{2b}\right)\right]\cdot I_{sc}^{2}=P_{sc}$$

where $r_{2}$, $r_{2b}$ are known (since they refer to Y windings) and $r_{1}\left(\delta \right)$ denotes the dependence expressed in Equation (9). It is straightforward to show that if the functional form r(δ) is substituted in Equation (12), the parameter δ is eliminated and thus may not be optimized. To bypass this situation, the following error function is defined:

(13) $$E\left(\delta \right)=P_{sc}-\left[\left(\frac{\alpha }{3}+\alpha_{b}\delta \right)r_{1}\left(\delta_{o}\right)+a^{2}\left(\alpha r_{2}+\alpha_{b}r_{2b}\right)\right]\cdot I_{sc}^{2}$$

where $\delta_{o}$ is a fixed value used to break the dependence with respect to Equation (9). At first, consider $\delta ,\delta_{o}\in R$. Since E(δ) is linear, given a domain $I_{\delta }=\left[\delta_{l},\delta_{h}\right]\subset R$, there must exist a set of $\delta_{o}$ values, henceforth denoted as $I_{\delta }^{o}=\left[\delta_{l}^{o},\delta_{h}^{o}\right]$, for which the error function has a zero for $\delta \in I_{\delta }$. Hence, δ can be optimized for a given $\delta_{o}$ by computing the zero $\delta^{*}\in I_{\delta }$ of Equation (13). Provided $I_{\delta }$ and $I_{\delta }^{o}$ are narrow (which is expected since δ≪1), $\delta^{*}$ can be obtained in a simple manner by discretizing $I_{\delta }$ and employing brute-force search. The procedure can then be repeated sequentially for other values of $\delta_{o}\in I_{\delta }^{o}$, yielding solution pairs $\left(\delta ,\delta_{o}\right)$. To arrive at a definite solution, it is simply proposed that $\delta_{o}=\frac{1}{2}\left(\delta_{l}^{o}+\delta_{h}^{o}\right)$ be selected. The corresponding resistance estimates are:

(14) $$r_{1}=\frac{3}{2+6\delta_{o}}r_{1\Delta }$$

(15) $$r_{1b}=\frac{3\delta }{2+6\delta_{o}}r_{1\Delta }$$

Please note that analogous considerations apply for the Δ-Δ connection, in which case optimization must be carried out over a linear function of two different δ parameters.

### 2.4. Summary of the Proposed Model

A brief summary of the proposed transformer model is given as follows. The DC resistance test imposes an unbalanced condition to the transformer terminals, for which reason a per-phase approach is unfeasible in this case. Hence, three-phase DC models are proposed for the Y and Δ windings, as seen in Figure 3 and Figure 4, respectively. Such DC models decompose transformer resistance into winding and contact components, thus providing greater detail than the standard model with respect to resistance distribution. For regular balanced operation, a per phase AC model as depicted in Figure 6 is proposed. It has the same topology as the exact model and incorporates the DC model resistances by means of temperature adjustment factors, which account for on-load heating. The only approximation maintained from the standard model is $x=\;x_{1}\approx a^{2}x_{2}$ per phase.

In particular, Figure 5 depicts the DC model of a Δ winding in open connection, for which an additional resistance measurement can be obtained. As discussed in Section 2.2 and Section 2.3, all DC and AC model parameters can be determined analytically if this measurement can be made, whereas an iterative approach is required otherwise.

### 2.5. On the Connection Resistance

Transformer connection resistance is often associated with the bushing conductors which precede the terminals [19]. However, it must be observed that the connection resistance parameter proposed in this work accounts for other resistance components in series with that of the bushing conductor, such as lead and contact resistance [20,21]. In fact, the proposed model may still be applied even if no bushing conductor exists (e.g., a low voltage transformer with borne connectors), in which case the proposed resistance parameters yield a measure of the remaining series resistance components.

## 3. Experiment

**Figure 7:**
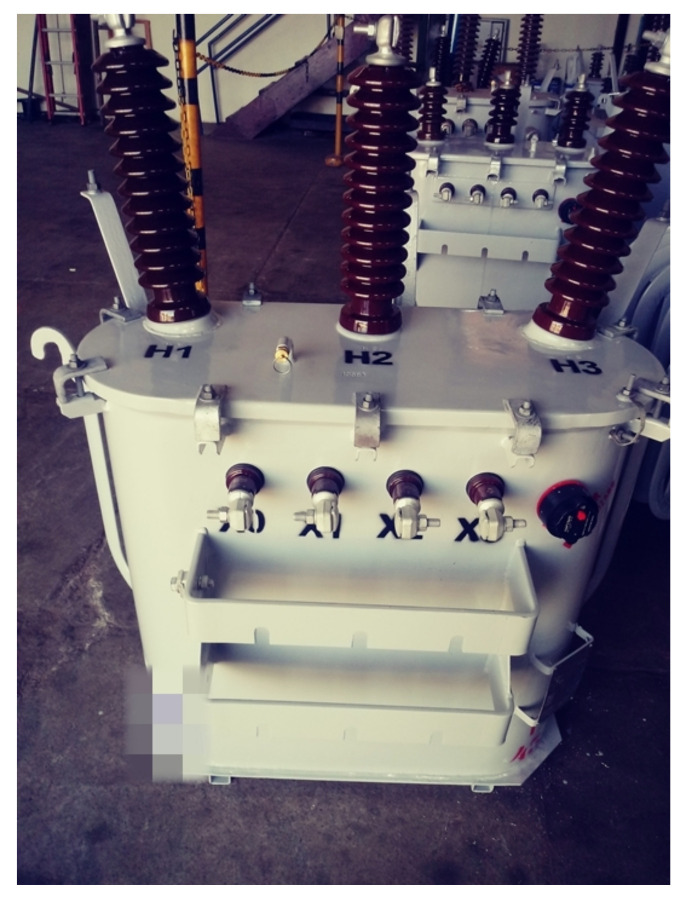
Picture of the transformer considered in the experiment.

To validate the proposed approach, an experiment was carried out to verify its performance in accurately estimating parameter values of the Δ-Y transformer with 34.5 kV:380 V voltage rating and 45kVA nominal power depicted in Figure 7.

Only ambient temperature measurements were available at the testing facility, where a rounded value of $T_{DC}=27{\;}^{\circ }C$ was obtained. It is assumed that windings at full load are, as per the nameplate, $50{\;}^{\circ }C$ above $T_{DC}$, hence $T=77{\;}^{\circ }C$. The transformer windings and contacts use aluminum conductors, for which $\gamma \approx 0.4\%{/}^{\circ }C$ [22]. Such values are used to compute the temperature-corrected AC model parameters with $\alpha =\alpha_{b}$. Each Δ winding presented a single accessible terminal, for which reason the proposed δ parameter approach was employed. By means of preliminary computations, it was verified that $\delta_{l}=\delta_{l}^{o}=0$, $\delta_{h}^{o}=0.025$ and $\delta_{h}=0.050$ satisfy the root condition, being thus adopted. Sets $I_{\delta }$ and $I_{\delta }^{o}$ were discretized in intervals of width ℓ=0.001. All obtained test measurements (i.e., DC resistance, short and open circuit) are given in Table 1 and Table 2.

**Figure 8:**
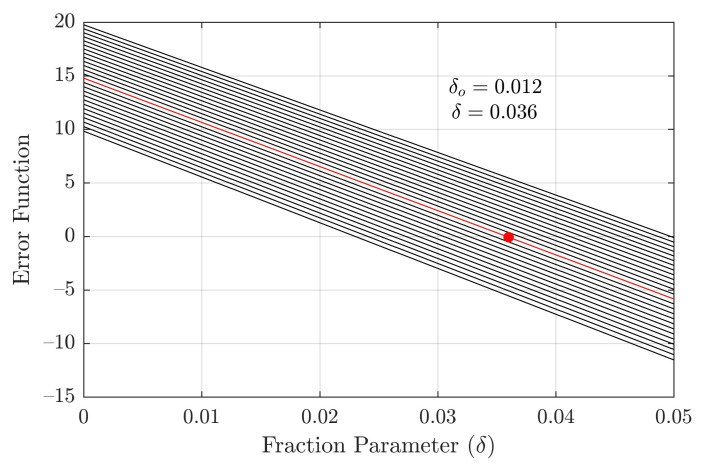
Plot of error function versus fraction parameter δ.

**Figure 9:**
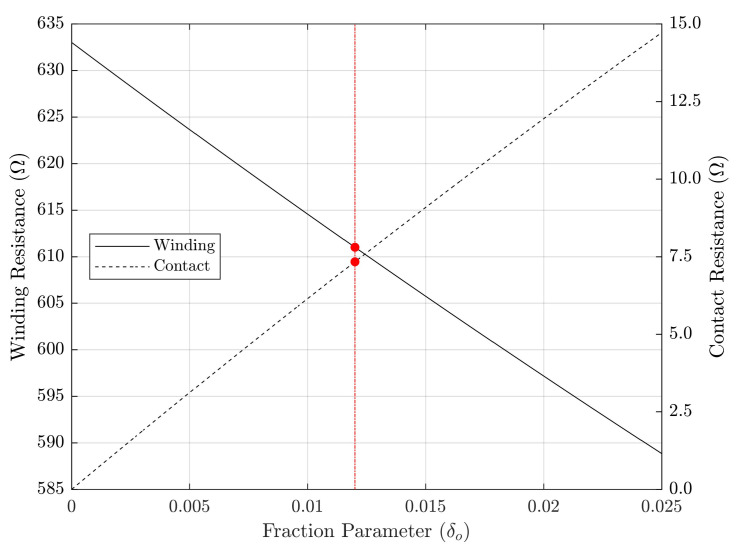
Plot of primary Δ resistances versus fraction parameter $\delta_{o}$.

A comparison between the obtained resistance parameter values and those yielded using solely short and open circuit tests to compute the standard transformer model is given in Table 3. The error function E(δ) is plotted in Figure 8 for the multiple values of $\delta_{o}\in I_{\delta }^{o}$, among which the selected value $\delta_{o}=\left\lfloor \frac{1}{2}\left(\delta_{l}^{o}+\delta_{h}^{o}\right)\right\rfloor =0.012$ is highlighted, where the symbol ⌊·⌋ designates floor rounding to the third decimal place. Finally, Figure 9 corresponds to the plots of solution pairs $\left(r_{1},r_{1b}\right)$ corresponding to each $\delta_{o}$, with that associated with the selected $\delta_{o}=0.012$ also being highlighted. Total computation time associated with the brute-force search in domains $I_{\delta }$ and $I_{\delta }^{o}$ was 1.24ms.

Please note that inductive and core loss parameter values have been omitted from Table 3 since their results are, as expected, identical to those of the standard method. At first, a qualitative analysis of the computed resistive parameters shows that the proposed method successfully separated total resistance into winding and contact components. In fact, as seen from Table 3, the temperature-corrected winding resistances reasonably approximate those of the standard model, which is expected due to their dominance with respect to contact resistances. Furthermore, the obtained contact resistances have small absolute values, which is also according to expectation. Such parameter features show that no divergence from the underlying physical problem occurs.

Let the superscript *SM* denote the standard model parameters. Consistency of the proposed model can also be inferred by noticing, from Table 3, that the approximate relations $r_{1}+3r_{1b}\approx r_{1b}^{SM}$ and $r_{2}+r_{2b}\approx r_{2b}^{SM}$ are valid. The factor-of-three difference in the contact resistance term is associated with the winding connection: since the primary is Δ-connected, the Y equivalent winding resistances are $\frac{1}{3}r_{1}$ and $\frac{1}{3}r_{1}^{SM}$ for the proposed and standard models, respectively. Furthermore, $r_{1b}$ is in series with $\frac{1}{3}r_{1}$, from which it is seen that $r_{1}+3r_{1b}\approx r_{1b}^{SM}$ must be approximately valid. On the other hand, the Y-connected secondary has its winding resistances directly in series with phase contact resistances, which makes the relation $r_{2}+r_{2b}\approx r_{2b}^{SM}$ approximately true.

Additional evidences regarding robustness and consistency of the proposed method are: (a) as seen in Figure 8 and Figure 9, feasible domains $I_{\delta }$ and $I_{\delta }^{o}$ correspond to small values of δ, as would be reasonably expected of contact resistance magnitude; (b) as seen in Figure 8, the actual existence of zeroes of the function E(δ) in $I_{\delta }$ indicates that the model is compatible with the physical measurements; and (c) the DC resistance values from Table 3 are discrepant from those of the standard model (which are referred to on-load temperature), but become strongly matched after temperature correction.

It is seen in Table 3 that $r_{1}^{SM}=a^{2}r_{2}^{SM}$ (in per phase values), which is a consequence of the $r=\;r_{1}\approx a^{2}r_{2}$ per phase approximation adopted in the standard model. This is not true for the proposed model, which shows that it captures the fact that transformer resistances are not perfectly balanced between primary and secondary in a per unit sense. Such detailing of resistance distribution is unattainable with the standard model.

Finally, application of the proposed method leads to the conclusion that transformer contact resistance is, in fact, *not* entirely negligible. This is seen to be especially true for the low voltage winding, in which contact resistance equalled 17.5% of the corresponding winding resistance. On the other hand, an analogous percentage of 1.2% was obtained for the high voltage winding. This is clearly due to small impedance of the low voltage winding, which leads to the expectation that contact resistance may be even more expressive for low voltage windings of transformers with higher power ratings.

## 4. Comparison of Output Voltage Computation

To further evaluate the proposed model, it is compared to the exact and standard models in terms of output voltage computation for varying load values. Consider first the parallel branch parameters, which are identical for all models. Such parameters were computed via open circuit test results (as discussed in Section 2.3). The obtained primary-referred values are $x_{m}=2.9\;M\Omega$ and $r_{m}=1.8\;M\Omega$. Now, for the proposed and standard models, the series resistive parameters are given in Table 3 and x=591.6Ω was obtained using Equation (11). It remains to characterize the series parameters of the exact model. As for the standard model, it is assumed that only open and short circuit data are available in this case. Since no additional measurements are available for computing the series parameters, we adopt the usual procedure [23] of assuming $r_{1}=K\cdot r_{tot}$ and $r_{2}=\left(1-K\right)\cdot r_{tot}$ per unit, K∈[0,1], where $r_{tot}$ is the total resistance, with an analogous assumption being made for the values of $x_{1}$ and $x_{2}$. In order to focus on more realistic parameter values, the exact model is evaluated for K∈[0.45,0.55].

**Figure 10:**
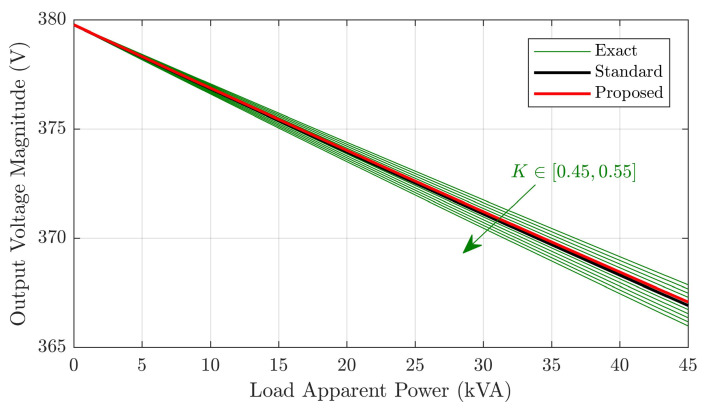
Output voltage magnitude versus load for the different models.

**Figure 11:**
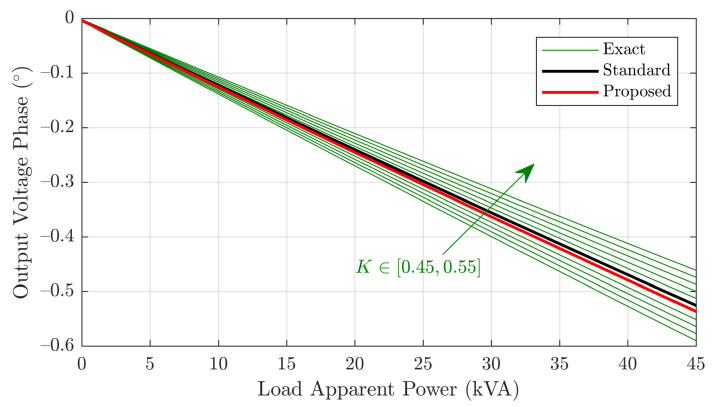
Output voltage phase versus load for the different models.

A load power factor of 0.92 (inductive) was considered, with apparent power being varied from zero to the rated transformer value of 45kVA. The resulting output voltage magnitudes and phases for each model are plotted in Figure 10 and Figure 11, respectively.

The results show that voltage magnitude and phase yielded by the proposed model are similar to those of the exact and standard models. This is an expected result, since series and parallel branch parameters tend to have relatively low and high magnitudes, respectively. The standard model fits best to the exact model with K=0.5, whereas the proposed one approaches the K=0.51 plots, which is due to the proposed model being able to incorporate unequal primary and secondary per unit impedances. In this sense, the matching for K=0.51 suggests the transformer primary has slightly greater per unit impedance, which is precisely what was obtained with the proposed model. In fact, it is easy to verify from Table 3 that the inequality $\frac{1}{3}r_{1}^{SM}/r_{2}^{SM}<\left(\frac{1}{3}r_{1}+r_{1b}\right)/\left(r_{2}+r_{2b}\right)$ between per phase impedance ratios is valid, which is consistent with K>0.5.

Hence, it is concluded that the proposed model is expected to have greater precision than the standard model, especially for cases in which significant mismatch between primary and secondary per unit impedance exists. For symmetrical transformers such as the one considered in this work, a slight precision improvement with respect to the standard model is obtained, which was confirmed by the matching between voltage magnitude and phase plots for the proposed model and exact model with K=0.51.

## 5. Conclusions

A novel low-frequency steady-state transformer model which separately accounts for contact and winding resistances was proposed. It consists of equivalent primary and secondary DC circuits and a per-phase AC model which incorporates the DC circuit resistances by means of temperature correction factors. Furthermore, a method was established for computing all model parameters by means of measurements acquired on usual transformer tests, namely DC resistance, short and open circuit tests. The model and method were validated via experiment on a real distribution transformer, whose results suggest the robustness and accuracy of the proposed approach. In fact, experimental results led to the following conclusions: (a) the proposed method enables computation of model parameter values via very fast brute-force search; (b) the model enables an accurate decomposition of transformer resistance into winding and contact components; and (c) the only additional data required for model computation are measurements from the DC resistance test, which is usually carried out in conjunction with the open circuit and short circuit tests. Finally, application of the proposed method also led to the interesting conclusion that contact resistance, especially that associated with low voltage windings, may not be entirely negligible in power transformers.

## Figures and Tables

**Table 1:** Open and short circuit test measurements.

Test	Voltage (V)	Current (A)	Power (W)
Short Circuit	1438	0.75	849
Open Circuit	380	0.91	183

**Table 2:** DC resistance test measurements.

Test	Resistance
$r_{1\Delta }$ (Ω)	422
$r_{2\Phi }$ (mΩ)	47
$r_{2\phi }$ (mΩ)	27

**Table 3:** Computed power transformer model parameters.

Parameter	Proposed Model (DC Resistances)	Proposed Model (Temperature Correction)	Standard Model
$r_{1}\;\left(\Omega \right)$	611.0	733.2	754.7
$r_{2}\;\left(m\Omega \right)$	20.0	24.0	30.4
$r_{1b}\;\left(\Omega \right)$	7.3	8.8	—
$r_{2b}\;\left(m\Omega \right)$	3.5	4.2	—

## Data Availability

The data presented in this study are available in the article.

## References

[1] Sereeter B., Vuik K., Witteveen C. (2017). Newton Power Flow Methods for Unbalanced Three-Phase Distribution Networks. Energies.

[2] Kersting W. (2016). Distribution System Modeling and Analysis.

[3] Sauter P.S., Braun C.A., Kluwe M., Hohmann S. Comparison of the Holomorphic Embedding Load Flow Method with Established Power Flow Algorithms and a New Hybrid Approach. Proceedings of the 2017 Ninth Annual IEEE Green Technologies Conference (GreenTech).

[4] Rao B.V., Kupzog F., Kozek M. (2019). Three-Phase Unbalanced Optimal Power Flow Using Holomorphic Embedding Load Flow Method. Sustainability.

[5] Chen T.H., Chen M.S., Inoue T., Kotas P., Chebli E. (1991). Three-phase cogenerator and transformer models for distribution system analysis. IEEE Trans. Power Deliv..

[6] Srithapon C., Fuangfoo P., Ghosh P.K., Siritaratiwat A., Chatthaworn R. (2021). Surrogate-Assisted Multi-Objective Probabilistic Optimal Power Flow for Distribution Network with Photovoltaic Generation and Electric Vehicles. IEEE Access.

[7] Zargar B., Monti A., Ponci F., Martí J.R. (2021). Linear Iterative Power Flow Approach Based on the Current Injection Model of Load and Generator. IEEE Access.

[8] Liu D., Liu L., Cheng H., Zhang S., Xin J. (2021). An Extended DC Power Flow Model Considering Voltage Magnitude. J. Mod. Power Syst. Clean Energy.

[9] Claeys S., Deconinck G., Geth F. (2021). Voltage-Dependent Load Models in Unbalanced Optimal Power Flow Using Power Cones. IEEE Trans. Smart Grid.

[10] Deng L., Sun Q., Jiang F., Wang S., Jiang S., Xiao H.X., Peng T. (2018). Modeling and Analysis of Parasitic Capacitance of Secondary Winding in High-Frequency High-Voltage Transformer Using Finite-Element Method. IEEE Trans. Appl. Supercond..

[11] Changjiang Z., Qian W., Huai W., Zhan S., Bak C.L. (2021). Electrical Stress on the Medium Voltage Medium Frequency Transformer. Energies.

[12] Liu X., Wang Y., Zhu J., Guo Y., Lei G., Liu C. (2016). Calculation of Capacitance in High-Frequency Transformer Windings. IEEE Trans. Magn..

[13] Umans S., Kingsley C. (2014). Fitzgerald & Kingsley's Electric Machinery.

[14] Valchev V., Van den Bossche A. (2018). Inductors and Transformers for Power Electronics.

[15] IEEE Xfrmrs & Reactors Working Group Guide for Diagnostic Field Testing of Fluid-Filled Power Transformers, Regulators, and Reactors. https://standards.ieee.org/standard/C57_152-2013.html.

[16] IEEE Temp Rise Test Above NP Rating Working Group Recommended Practice for Performing Temperature Rise Tests on Liquid-Immersed Power Transformers at Loads Beyond Nameplate Ratings. https://standards.ieee.org/standard/C57_119-2018.html.

[17] Chowdhury R., Rusicior M., Vico J., Young J. How transformer DC winding resistance testing can cause generator relays to operate. Proceedings of the 2016 69th Annual Conference for Protective Relay Engineers (CPRE).

[18] Fehr R. (2015). Industrial Power Distribution.

[19] Hitachi ABB Power Grids (2020). Transformer bushing type GOB—Installation and maintenance guide. 2750 515-12 EN REV. 12.

[20] Gonzalez D., Hopfeld M., Berger F., Schaaf P. (2018). Investigation on Contact Resistance Behavior of Switching Contacts Using a Newly Developed Model Switch. IEEE Trans. Components, Packag. Manuf. Technol..

[21] Zhai C., Hanaor D., Proust G., Gan Y. (2017). Stress-Dependent Electrical Contact Resistance at Fractal Rough Surfaces. J. Eng. Mech..

[22] Giancoli D. (2008). Physics.

[23] De Leon F., Farazmand A., Joseph P. (2012). Comparing the T and *π* Equivalent Circuits for the Calculation of Transformer Inrush Currents. IEEE Trans. Power Deliv..

